# Atrial fibrillation is not uncommon among patients with ischemic stroke and transient ischemic stroke in China

**DOI:** 10.1186/s12883-017-0987-y

**Published:** 2017-12-04

**Authors:** Xiaomeng Yang, Shuya Li, Xingquan Zhao, Liping Liu, Yong Jiang, Zixiao Li, Yilong Wang, Yongjun Wang

**Affiliations:** 1 Vascular Neurology, Department of Neurology, Beijing Tiantan Hospital, Capital Medical University, No. 6 Tiantan Xili, Dongcheng District, Beijing, 100050 China; 2 Tiantan Clinical Trial and Research Center for Stroke, Department of Neurology, Beijing Tiantan Hospital, Capital Medical University, No.6 Tiantanxili, Dongcheng District, Beijing, 100050 China; 3 Center of Stroke, Beijing Institute for Brain Disorders, Beijing, China; 4 Neuro-intensive Care Unit, Department of Neurology, Beijing Tiantan Hospital, Capital Medical University, Beijing, China; 5 Beijing Key Laboratory of Translational Medicine for Cerebrovascular Disease, No. 6 Tiantan Xili, Dongcheng District, Beijing, 100050 China; 6 China National Clinical Research Center for Neurological Diseases, No. 6 Tiantan Xili, Dongcheng District, Beijing, 100050 China

**Keywords:** Atrial fibrillation, Prolonged ECG monitoring, ischemic stroke, transient ischemic attack, China

## Abstract

**Background:**

Atrial fibrillation (AF) is reported to be a less frequent cause of ischemic stroke in China than in Europe and North America, but it is not clear whether this is due to underestimation. Our aim was to define the true frequency of AF-associated stroke, to determine the yield of 6-day Holter ECG to detect AF in Chinese stroke patients, and to elucidate predictors of newly detected AF.

**Methods:**

Patients with acute ischemic stroke or transient ischemic attack (TIA) were enrolled in a prospective, multicenter cohort study of 6-day Holter monitoring within 7 days after stroke onset at 20 sites in China between 2013 and 2015. Independent predictors of newly-detected AF were determined by multivariate analysis.

**Results:**

Among 1511 patients with ischemic stroke and TIA (mean age 63 years, 33.1% women), 305 (20.2%) had either previously known (196, 13.0%) or AF newly-detected by electrocardiography (53, 3.5%) or by 6-day Holter monitoring (56/1262, 4.4%). A history of heart failure (OR = 4.70, 95%CI, 1.64–13.5), advanced age (OR = 1.06, 95%CI, 1.04–1.09), NIHSS at admission (OR = 1.06, 95%CI, 1.02–1.10), blood high density lipoprotein (HDL) (OR = 1.52, 95%CI, 1.09–2.13), together with blood triglycerides (OR = 0.64, 95%CI, 0.45–0.91) were independently associated with newly-detected AF.

**Conclusions:**

Contrary to previous reports, AF-associated stroke is frequent (20%) in China if systemically sought. Prolonged noninvasive cardiac rhythm monitoring importantly increases AF detection in patients with recent ischemic stroke and TIA in China. Advanced age, history of heart failure, and higher admission NIHSS and higher level of HDL were independent indicators of newly-detected AF.

**Trial registration:**

NCT02156765 (June 5, 2014).

## Background

The fraction of strokes associated with atrial fibrillation has been reported to be lower in China than in European and North American stroke populations [1–3], but it is not clear whether this is due to its under-detection and hence leading to suboptimal secondary prevention. Recent studies have demonstrated that prolonged (> 24 h) of cardiac rhythm monitoring importantly increases detection of paroxysmal atrial fibrillation in post-stroke patients [4–6], but this has not previously been assessed in Chinese stroke patients, who are on average relatively young. We sought to determine the true frequency of AF-associated stroke and yield of prolonged cardiac rhythm monitoring to detect paroxysmal AF in Chinese patients with recent ischemic stroke and TIA.

## Methods

### Subjects

From October 2013 to June 2015, we recruited consecutive patients within 7 days of the index event of ischemic stroke or TIA from 20 Chinese hospitals. Clinical TIAs with an acute ischemic lesion visualized on computerized tomography (CT) or magnetic resonance imaging (MRI) were classified as strokes. The exclusionary criteria were 1) age < 18 years; 2) patients who were unable or unwilling to give informed consent; and 3)patients who were found not to have a stroke or TIA (stroke mimics). The study complied with the Declaration of Helsinki, and protocol was approved by the institutional review board (IRB) of Beijing Tiantan Hospital, Capital Medical University. All patients or their legal representatives provided written informed consent.

### Data collection

Clinical data included demographic information, stroke risk factors, detailed medical history and treatments. Stroke severity was assessed using the National Institutes of Health Stroke Scale (NIHSS) [7] assessed by physicians. Patients received routine stroke care according to his/her condition including CT or MRI, 12-channel surface electrocardiography (ECG), transthoracic echocardiography, ultrasound of the cervical arteries, and routine blood analysis including, for example, lipid profile, blood glucose, liver enzymes and creatinine. Previously known AF was diagnosed according to the medical history reported by the patients and the prior medical records of the patients in our study.

### Holter monitoring

The 6-day Holter monitoring initiated within 7 days after the index event using a commercially available 3-lead monitor device (iHolter, Yocaly Information Science & Technology Co., Ltd. Jinan, Shandong, China). It is an autotriggered device that uses an event detection algorithm to capture asymptomatic paroxysmal events, including paroxysmal AF. The event detection algorithm uses RR interval variability and QRS morphology analysis to detect all possible AF events that are then transmitted to a central core laboratory for manual review and confirmation based on standard diagnostic criteria (absence of p wave activity and irregular RR interval). ECG recordings were analyzed in a central core laboratory by two investigators who were independent of our study team and who were unaware of clinical and neuroimaging results using dedicated analysis software (DoctorClient, Software Version 1.5.0.16). All Holter ECG recordings with suspected AF were subsequently evaluated by another independent observer. Bursts of AF on ECG were reported by the number of beats of each occurrence. The first time and longest duration of bursts of AF for each patient were also recorded. The device defined AF as ≥ 1 period of > 30 s duration of an absolute arrhythmia without detectable P-waves and episodes < 30 s require manual review of all possible AF events [8, 9]. Atrial flutter and AF were not discriminated as both arrhythmias carry a similar stroke risk and often coincide [10].

### Statistical analyses

Continuous data are given as median, range and categorical variables are given as absolute number and percentage. Mann–Whitney U test (continuous variables) and Pearson χ2 tests (categorical variables) were used to compare groups. Significance was assumed when the 2-sided probability value was <0.05. A multivariate analysis was performed using logistic regression, all factors that associated with detected AF in univariate analysis were included (*P*< 0.05). For calculation of 95% confidence interval (CI), binominal distribution was assumed. Statistical analysis was performed using SAS version 9.2 (SAS Institute Inc., Cary, NC).

## Results

### Patient population

**Fig. 1:**
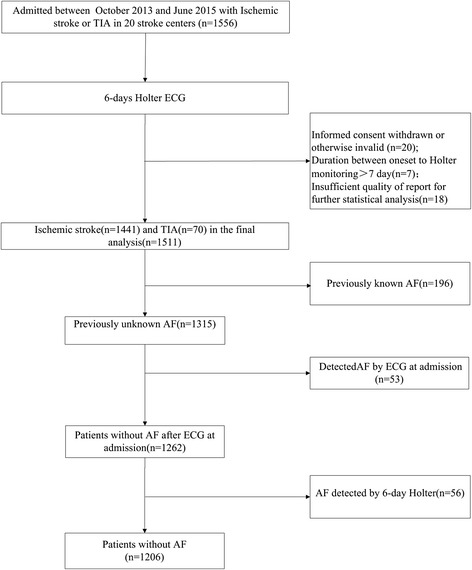
Flow chart of the study

During the study period, 1556 patients with stroke or TIA were admitted to the participating stroke units. Because of artifacts, interruptions for clinical procedures, and early detachment of the monitoring leads by patients, 1511 patients (1441 with ischemic stroke and 70with TIA) were included (Fig. 1). The mean age of the study cohort was 63 years, and 33.1% were women.

### Detection of Atrial fibrillation

**Fig. 2:**
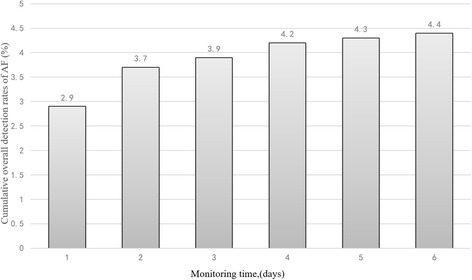
Cumulative overall detection rates of 56 unsuspected AF detected by 6-day Holter monitoring

Among 1511 patients with ischemic stroke and TIA, 305 (20.2%) had previously known ((196, 13.0%) or newly detected AF on electrocardiography (53, 3.5%), and 4.4% of patients had unsuspected AF detected by 6-day Holter monitoring (56/1262). The median duration from index event (TIA/stroke) to initiation of monitoring was 4 days (range, 2–5 days). Prolonged Holter monitoring was well tolerated, yielding a median ECG recording duration of 5 days (range, 5–6 days). Cumulative overall detection rates of 56 unsuspected AF detected by 6-day Holter monitoring were shown in Fig. 2. Patients with TIA/stroke frequently revealed other significant dysrhythmias, including 1) premature atrial complexes (1099,72.7%); 2) premature ventricular complexes (1123, 74.3%); 3) atrial tachycardia (728, 48.2%);4) ventricular tachycardia (142, 9.4%).

### Patient characteristics

Cohort characteristics, comparing patients with and without AF, are presented in Table 1. Older patients (median 72 vs. median 61; *P* < 0.001) and patients who are not current smokers (81.0% vs. 63.8%; *P* < 0.001) were more likely to have AF. Patients with AF were presented more frequently with previous ischemic stroke (27.2% vs. 21.1%; *P* = 0.02), coronary heart disease (17.7% vs. 8.3%; *P* < 0.001), heart failure (9.5% vs. 1.0%; *P* < 0.001) and higher NIHSS score at admission (median 4 vs. 3; P < 0.001). Considering laboratory tests, patients with AF had, on average, lower levels of triglycerides (median 1.05 vs. 1.40; *P* < 0.001) and LDL (median 2.57 vs. median 2.74; *P* = 0.001) and cholesterol (median 4.35 vs. median 4.55; *P* = 0.002)) and had higher levels of HDL (median1.20 vs. 1.12; *P* < 0.001).

**Table 1:** Patient features associated with AF

Variables	All patients (*n* = 1511)	Without AF (*n* = 1206)	With AF (*n* = 305)	*P* Value
Age,yr. (median[IQR])	63(55–73)	61(53–71)	72(64–78)	< 0.001
Women, n(%)	500(33.1)	385 (31.9)	115(37.7)	0.06
BMI,Kg/m^2^ (median[IQR])	24.2(22.0–26.4)	24.2(22.0–26.4)	24.2(21.5–26.3)	0.08
Current smoker,n(%)	494(32.7)	436(36.2)	58(19.0)	< 0.001
Medical history, (%)				
Hypertension	949(62.8)	750 (62.2)	199 (65.2)	0.32
Diabetes	326(21.6)	254 (21.1)	72 (23.6)	0.33
Hyperlipidemia	227(15.0)	192 (15.9)	35(11.5)	0.052
Ischemic stroke	337(22.3)	254 (21.1)	83 (27.2)	0.02
TIA	43(2.8)	36 (3.0)	7(2.3)	0.52
MI	45(3.0)	33 (2.7)	12 (3.9)	0.27
Coronary heart disease	154(10.2)	100(8.3)	54(17.7)	< 0.001
Peripheral arterial disease	32(2.1)	23(1.9)	9(3.0)	0.26
Heart failure	41(2.7)	12 (1.0)	29(9.5)	< 0.001
Blood analysis(mmol/L)				
Triglycerides	1.33(0.90–1.91)	1.40(0.96–2.00)	1.05(0.78–1.56)	< 0.001
LDL	2.70(2.20–3.31)	2.74(2.23–3.35)	2.57(2.05–3.22)	0.001
HDL	1.12(0.96–1.34)	1.12(0.96–1.34)	1.20(1.00–1.41)	0.002
Cholesterol	4.52 (3.90–5.24)	4.55(3.93–5.29)	4.35(3.76–5.06)	0.002
Fasting blood-glucose	5.49(4.92–6.81)	5.47(4.92–6.81)	5.60(4.94–6.83)	0.42
Clinical features(median[IQR])				
NIHSS at admission	3(1–6)	3(1–6)	4(2–9)	< 0.001

*IQR* indicates interquartile range, *TIA* transient ischemic attack, *MI* myocardial infarction, *NIHSS* the National Institutes of Health Stroke Scale, *LDL* low density lipoprotein, *HDL* high density lipoprotein

In multivariable analysis (Table 2), a history of coronary heart disease (OR = 1.65, 95%CI, 1.08–2.50) and heart failure (OR = 7.11,95%CI,3.22–15.72), advanced age (OR = 1.06, 95%CI, 1.05–1.08), admission NIHSS (OR = 1.06, 95%CI, 1.04–1.09), HDL (OR = 1.41, 95%CI, 1.02–1.94) together with current smoker (OR = 0.68, 95%CI, 0.48–0.97) and blood triglycerides (OR = 0.78, 95%CI, 0.65–0.94) were independently associated with AF in patients with ischemic stroke and TIA.

**Table 2:** Multivariate analysis of clinical features associated with AF

Variables	OR	95% CI	*P* value
Age	1.06	1.04–1.08	< 0.001
Previous coronary heart disease	2.00	1.31–3.05	0.001
Previous heart failure	7.36	3.38–16.05	< 0.001
NIHSS at admission	1.05	1.03–1.08	< 0.001
Current smoker	0.65	0.45–0.93	0.02
Triglycerides	0.76	0.63–0.91	0.004

NIHSS indicates the National Institutes of Health Stroke Scale

We also compared the characteristics of patients with previously known/ newly-detected AF and without AF (Table 3). Older patients (median 72 vs. median 61; *P* < 0.001) and patients who are not current smokers (77.1% vs. 63.8%; *P* = 0.006) were more likely to have newly-detected AF. Patients with newly-detected AF were presented more frequently with previous TIA (6.4% vs. 1.0%; *P* < 0.001), coronary heart disease (17.4% vs. 8.3%; *P* = 0.001), heart failure (6.4% vs. 1.0%; P < 0.001), higher NIHSS score at admission (median 4 vs. 3; *P* < 0.001) and less frequently with previous hyperlipidemia (8.3% vs. 15.9%; *P* = 0.03). Considering laboratory tests, patients with newly-detected AF had, on average, lower levels of triglycerides (median 1.03 vs. 1.40; *P* < 0.001) and cholesterol (median 4.38 vs. median 4.55; *P* = 0.05)) and had higher levels of HDL (median1.22 vs. 1.12; *P* = 0.002).

**Table 3:** Patient characteristics with previously known, newly detected AF or without AF

Variables	Without AF (*n* = 1206)	Previously known AF (*n* = 196)	Newly detected AF (*n* = 109)	*P* Value^*^	*P* Value^†^
Age, yr. (median[IQR])	61(53–71)	72(64–78)	72(64–78)	< 0.001	< 0.001
Women, n (%)	385(31.9)	75(38.3)	40 (36.7)	0.08	0.31
BMI, Kg/m^2^ (median[IQR])	24.2(22.0–26.4)	24.2(21.3–26.4)	23.8(21.9–25.6)	0.27	0.11
Current smoker, n (%)	436(36.2)	33(16.8)	25(22.9)	< 0.001	0.006
Medical history, (%)					
Hypertension	750(62.2)	136(69.4)	63 (57.8)	0.053	0.37
Diabetes	254(21.1)	46(23.5)	26 (23.9)	0.45	0.50
Hyperlipidemia	192(15.9)	26(13.3)	9(8.3)	0.34	0.03
Ischemic stroke	254(21.1)	67(34.2)	16 (14.7)	< 0.001	0.11
TIA	12(1.0)	6(3.1)	7 (6.4)	0.95	< 0.001
MI	33(2.7)	7(3.6)	5(4.6)	0.52	0.42
Coronary heart disease	100(8.3)	35(17.9)	19 (17.4)	< 0.001	0.001
Peripheral arterial disease	23(1.9)	8(4.1)	1(0.9)	0.10	0.71
Heart failure	12(1.0)	22(11.2)	7(6.4)	< 0.001	< 0.001
Blood analysis (mmol/L)					
Triglycerides	1.40(0.96–2.00)	1.05(0.75–1.60)	1.03(0.81–1.48)	< 0.001	< 0.001
LDL	2.74(2.23–3.35)	2.49(2.03–3.25)	2.65(2.13–3.17)	0.003	0.07
HDL	1.12(0.96–1.34)	1.19(0.99–1.38)	1.22(1.02–1.45)	0.06	0.002
Cholesterol	4.55(3.93–5.29)	4.28(3.73–5.14)	4.38(3.77–4.94)	0.01	0.05
Fasting blood-glucose	5.47(4.92–6.81)	5.63(4.98–7.10)	5.50(4.87–6.51)	0.18	0.67
Clinical features (median[IQR])					
NIHSS at admission	3(1–6)	4(2–9)	4(2–10)	< 0.001	< 0.001

*IQR* indicates interquartile range, *TIA* transient ischemic attack, *MI* myocardial infarction, *NIHSS* the National Institutes of Health Stroke Scale, *LDL* low density lipoprotein, *HDL* high density lipoprotein

*P values for comparation between patients with previously known AF and those without AF

†P values for comparation between patients with newly-detected AF and those without AF

In multivariable analysis (Table 4), a history of heart failure (OR = 4.70, 95%CI, 3.22–15.72), advanced age (OR = 1.06, 95%CI, 1.04–1.09), admission NIHSS (OR = 1.06, 95%CI, 1.02–1.10), HDL (OR = 1.52, 95%CI, 1.09–2.13) together with blood triglycerides (OR = 0.64, 95%CI, 0.45–0.91) were independently associated with newly-detected AF.

**Table 4:** Multivariate analysis of clinical features independently predictive of newly detected AF

Variables	OR	95% CI	*P* value
Age	1.06	1.04–1.09	< 0.001
Previous heart failure	4.70	1.64–13.5	0.004
NIHSS at admission	1.06	1.02–1.10	0.003
Triglycerides	0.64	0.45–0.91	0.01
HDL	1.52	1.09–2.13	0.02

*NIHSS* the National Institutes of Health Stroke Scale, *LDL* low density lipoprotein, *HDL* high density lipoprotein

## Discussion

Contrary to previous reports, AF-associated brain ischemia is frequent (20.2%) in China. Identification of AF after ischemic stroke or TIA is crucial to optimize appropriate anticoagulation. This is the first study to demonstrate the yield of prolonged cardiac rhythm monitoring after ischemic stroke and TIA in Chinese patients with recent brain ischemia. This prospective multicenter study provides solid evidence that AF is considerably more common in patients with ischemic stroke and TIA than what has been reported in most previous population-based studies in China [2, 3, 11]. The underlying reasons are likely the increased duration of cardiac rhythm monitoring in comparison to prior studies and AF detection based upon a standardized automated arrhythmia detection algorithm and continuous review of monitoring data.

However, when compared with the studies in western countries [12, 13], the overall detection rate of AF among stroke and TIA is still lower. In our study the detection rate of any new AF among patients without previously known AF were respectively 5.2% and 6.9% in the first 24 h and during the whole monitoring period while the rate were 6.2% and 14.1% in a recent review [14]. The discrepancy might be because the patients in our study are, on average younger (mean age, 63.0 years) than in studies reporting higher frequencies AF in which the mean ages was 68.4 years. When compared with studies with the similar monitoring duration, Stahrenberg et al. [12]and Jabaudon et al. [15] have reported higher detection rates of new AF using 7-day continuous Holter monitoring and 7-day event-loop recording (ELR) device (12.5% and 14.8% respectively). The early time point of monitoring after symptom onset (9.5 h) and relatively older patients in their studies may be the explanation for the difference.

In our study, we found that patients with history of previous coronary heart disease have a higher likelihood of AF which is consistent with previous study [16]. We suppose that the explanation might be that coronary heart disease shares similar risk factors such as aging and systemic vascular risk factors with AF which may cause an abnormal atrial tissue substrate or atrial cardiopathy, that can result in AF.

We also found that current smoker was less likely to have AF in our study which is in accordance with previous study [13]. However, this is inconsistent with recent study investigating the association between smoking and AF in community population which have shown smoking was associated with the incidence of AF [17]. We speculate that the inconsistency may lie in the different population. As we known smoking is an important and potent risk factor for atherosclerosis which may be the main reason for stroke in patients without AF, accordingly the percentage of current smoker is higher in stroke patients without AF. Prospective studies about the association between smoking and AF in stroke patients are needed in the future.

Several factors were significantly associated with an increased risk of new AF detection, namely older age, NIHSS at admission and previous heart failure and HDL. Our results are in line with previous studies in which advanced age was shown to be associated with a higher incidence of AF and paroxysmal atrial fibrillation (pAF) after stroke [16, 18, 19]. This argues for a structural abnormality of the atria with age which may lead to electrical instability thus causing pAF. As cardioembolic strokes are frequently larger and associated with an adverse outcome than those due to other sources [20], we also found that higher NIHSS score at admission is an independent predictor of detection of AF.

In addition, we found that patients with history of heart failure to have a higher likelihood of newly detected AF which is in accordance with previous studies [16, 21]. Previous study also showed preexisting heart disease is the major cause of AF that is first diagnosed after stroke [21], so more intensified screening for AF in these patients may be essential and meaningful.

We also observed independent association between blood triglycerides/HDL and detected AF. The association between blood lipids and AF risk has been reported by several previous publications which have offered inconsistent results [22]. Lack of adjustment for important confounders and inclusion of lipid-lowering medication users may affect the finding in our study which should be explained in caution.

There are limitations to our study: firstly, patients enrolled in our study had, on average, relatively mild strokes which enabled them to complete the study protocol. As cardioembolism is likely to cause large, severe strokes, the detection rate of AF may be underestimated in our study. Secondly, because our analysis focused on the usefulness of Holter monitoring on detection of AF, detailed diagnostic workup such as transesophageal echocardiography or additional biomarkers were only completed in some patients. This limited the predictive model regarding clinical indicators that be associated with decreased risk of AF. Thirdly, some of the brief occurrences of AF may represent stroke-induced arrhythmias rather than being the cause of the stroke. However, whether pAF occurred before or after the stroke will always be difficult to clarify because prestroke monitoring is not available.

## Conclusion

In conclusion, we found that AF is more common among patients with ischemic stroke and TIA in China than those previously reported. For the first time in a Chinese population, we have demonstrated that AF can be detected in an important fraction of patients with ischemic stroke and TIA via prolonged monitoring evaluation. Clinicians should plan the best available combination of post-stroke atrial fibrillation screening methods to increase yield of AF detection in the shortest possible time after stroke or TIA in order to optimize secondary antithrombotic management.

## Abbreviations

AF: Atrial fibrillation
CI: Confidence interval
CT: Computerized tomography
ELR: Event-loop recording
HDL: High density lipoprotein
IQR: Interquartile range
LDL: low density lipoprotein
MI: Myocardial infarction
MRI: Magnetic resonance imaging
NIHSS: National Institutes of Health Stroke Scale
OR: Odds-Ratio
pAF: Paroxysmal Atrial fibrillation
TIA: Transient ischemic attack
